# Ventricular Arrhythmias: Epidemiology, Diagnosis and Treatment

**DOI:** 10.3390/jcdd13090458

**Published:** 2026-09-11

**Authors:** Antonio Di Monaco, Massimo Grimaldi

**Affiliations:** 1 Department of Cardiology, General Regional Hospital “F. Miulli”, Acquaviva delle Fonti, 70021 Bari, Italy; m.grimaldi@miulli.it; 2 Department of Medicine and Surgery, LUM University, Casamassima, 70021 Bari, Italy

Ventricular arrhythmias (VAs) represent a broad and clinically heterogeneous spectrum of cardiac rhythm disturbances, ranging from isolated ventricular premature beats to life-threatening ventricular fibrillation (VF) and sudden cardiac death (SCD). VAs occur most frequently in the context of structural heart disease; however, a significant minority of patients present with idiopathic VA in the absence of identifiable cardiac pathology [1]. The epidemiological profile of VA varies markedly by age: in younger patients, channelopathies, cardiomyopathies, myocarditis, and substance abuse predominate, whereas in older populations, chronic degenerative conditions, including coronary artery disease, valvular heart disease and heart failure, are the principal underlying substrates [1].

Refractory unstable VAs and electrical storms are associated with mortality rates as high as 50%, underscoring their status as a major public health burden [1]. Pharmacological therapy is often insufficient to control recurrent arrhythmias and carries well-recognized limitations, including pro-arrhythmic effects and end-organ toxicity. Catheter ablation has emerged as an important pillar of management, capable of reducing recurrent VA episodes and improving patient prognosis; nevertheless, procedures in hemodynamically unstable patients carry significant risks of procedural complications and mortality [1]. The integration of mechanical circulatory support during ablation has shown promise in mitigating peri-procedural adverse events [1,2,3].

In recent years, remarkable advances have been achieved in both the diagnosis and treatment of VAs. Advanced imaging modalities, including computed tomography (CT) and cardiac magnetic resonance (CMR), provide unprecedented characterization of arrhythmogenic substrate, while novel catheter ablation techniques and stereotactic radioablation have expanded the therapeutic strategies [1,4,5]. Genetic testing has further refined risk stratification, particularly in inherited channelopathies and cardiomyopathies, enabling earlier identification of high-risk individuals [6,7].

The aim of this Special Issue of the *Journal of Cardiovascular Development and Disease* (*JCDD*), titled “Ventricular Arrhythmias: Epidemiology, Diagnosis, and Treatment”, is to provide a clear and modern overview of contemporary VA management, with a focus on advanced imaging and therapeutic techniques. The eight papers assembled here span innovative biomarker research, arrhythmia characterization in specific cardiomyopathies, advanced imaging for risk stratification, novel ablation strategies, and device-based therapies, collectively advancing the field.

Cetin and colleagues (Contribution 1) present a retrospective pilot study investigating the relationship between plasma apelin levels and sustained VT in patients with heart failure with reduced ejection fraction (HFrEF). Ninety patients were divided into three groups: HFrEF with documented VT, HFrEF without VT, and controls. Apelin levels were significantly lower in both HFrEF groups compared to controls and lowest in those with documented VT; each 1 ng/mL decrease in apelin was independently associated with a 69% increase in the odds of VT (OR 0.313; 95% CI: 0.124–0.788; *p* = 0.014) (Contribution 1). A cut-off of 0.80 ng/mL showed 77% sensitivity and 75% specificity for VT discrimination. This work identifies apelin as a novel neurohormonal biomarker for arrhythmia risk stratification in HFrEF, complementing established structural and functional parameters, and opens a pathway for prospective validation in larger multicenter cohorts.

Güler-Eren and colleagues (Contribution 2) contribute a retrospective analysis of 104 consecutive patients with Takotsubo syndrome (TTS) from a large single-center registry, examining the relationship between early arrhythmias and long-term adverse cardiac outcomes over a median follow-up of 2.1 years. Ventricular arrhythmias occurred in 15.4% of patients during index hospitalization and emerged as the strongest independent predictor of adverse events (OR 3.94, 95% CI: 1.22–12.69; *p* = 0.021), outperforming QTc prolongation and left ventricular dysfunction (Contribution 2). This association persisted after adjusting for medical triggers. These findings challenge the perception of TTS as a universally benign and transient cardiomyopathy and mandate that patients presenting with early VAs undergo prolonged rhythm monitoring and structured follow-up.

Aljehani and colleagues (Contribution 3) present a retrospective cohort study of 83 patients with arrhythmogenic right ventricular cardiomyopathy (ARVC), evaluating the prognostic value of right ventricular free wall longitudinal strain (RVFWLS) by two-dimensional speckle-tracking echocardiography. Over three years of follow-up, RVFWLS was the only independent predictor of major adverse cardiac events (MACE)—defined as sustained VT, VF, appropriate ICD therapy, heart failure, cardiac transplantation, or cardiac death—outperforming conventional parameters including RV fractional area change and TAPSE (HR 1.4, 95% CI: 1.0–2.0; *p* = 0.026) (Contribution 3). LV global longitudinal strain also independently predicted MACE, underscoring the biventricular nature of ARVC pathology. This study supports the integration of strain imaging into routine ARVC assessment as a refined tool for identifying patients who may benefit from ICD implantation or intensified therapy.

Yousefli and colleagues (Contribution 4) provide a comprehensive narrative review synthesizing current evidence on prophylactic versus reactive VT ablation in repaired tetralogy of Fallot (rTOF), in which VT and SCD remain the principal late causes of mortality. The review delineates the anatomical basis of VT, predominantly macroreentrant circuits involving four scar-related isthmuses, with Isthmus 3 (between the VSD patch and pulmonary annulus) implicated in 80–90% of inducible VTs and critically appraises the emerging shift from reactive to proactive substrate ablation (Contribution 4). Proactive ablation prior to clinical arrhythmia has shown potential to reduce ICD eligibility from 25–51% to 11% in single-center data, while reactive ablation achieves pooled VT recurrence reduction (RR 0.11; 95% CI: 0.03–0.33) (Contribution 4). The ongoing CATAPULT-TOF trial will provide the first multicenter prospective evidence on preemptive substrate ablation, particularly in patients scheduled for pulmonary valve replacement.

Guarracini and colleagues (Contribution 5) provide a state-of-the-art review of stereotactic arrhythmia radioablation (STAR) for refractory VT, covering technical foundations, patient selection, safety, and evolving indications. STAR employs multimodal imaging (CT, late gadolinium enhancement CMR, electroanatomic mapping, and noninvasive electrocardiographic imaging) to accurately localize arrhythmogenic substrate and delivers a single-fraction dose of 25 Gy to induce fibrotic remodeling non-invasively (Contribution 5). While initial data demonstrate meaningful VT burden reduction in patients refractory to medication and conventional ablation, the adverse event profile, including short-term cardiac decompensation and long-term risks of pericarditis, pneumonitis, and coronary injury, demands rigorous patient selection. The authors position STAR as a palliative option for patients with truly refractory VT and no viable alternatives, requiring multidisciplinary expertise available only in specialized centers.

Sun and colleagues (Contribution 6) report the first pediatric application of an extravascular implantable cardioverter-defibrillator (EV-ICD) in a 9-year-old child with congenital long QT syndrome type 2 (cLQTS2) and a prior out-of-hospital cardiac arrest, harboring a pathogenic KCNH2 mutation. Despite maximal pharmacological therapy, the patient remained at very high SCD risk. The EV-ICD was implanted with meticulous preoperative CT-guided anatomical planning and intraoperative provision of lead slack to accommodate somatic growth. At 2-month follow-up, device parameters remained satisfactory with no complications or inappropriate shocks (Contribution 6). This case report highlights the unique advantages of the EV-ICD in children: preservation of venous access, avoidance of transvenous lead complications related to growth, and antitachycardia pacing capability.

Romanazzi and colleagues (Contribution 7) describe an illustrative case of STAR guided by the CardioInsight noninvasive electrocardiographic imaging (ECGI) system in a patient with ischemic refractory VT and persistent LV apical thrombosis, which entirely precluded conventional catheter ablation. The 252-electrode vest combined with CT-derived epicardial geometry non-invasively mapped two induced VTs, identifying the middle septum and anterior LV wall as the arrhythmogenic targets (Contribution 7). Treatment was delivered via a TrueBeam linear accelerator with image-guided and surface-guided radiotherapy, lasting only three minutes without acute complications. At one-year follow-up, a significant reduction in arrhythmia burden was documented with no recurrent VT. This case advances the concept of a fully non-invasive mapping-to-ablation workflow for patients with contraindications to invasive procedures and underscores the synergy between ECGI and STAR in overcoming the technical limitations of conventional approaches.

Stankowski and colleagues (Contribution 8) present a paradigmatic case report illustrating the pivotal role of CMR in SCD risk stratification in hypertrophic cardiomyopathy (HCM). A 24-year-old man with apical HCM underwent comprehensive multiparametric CMR (native T1 and T2 mapping, extracellular volume (ECV) quantification, late gadolinium enhancement (LGE), and feature-tracking strain analysis) alongside genetic testing that identified a pathogenic MYBPC3 variant. CMR revealed extensive LGE encompassing 28% of LV mass, elevated native T1 and T2 values, and increased ECV (42%) (Contribution 8). Despite an intermediate ESC 5-year SCD risk score and the absence of all five conventional AHA/ACC major risk factors, the extent of LGE upgraded the indication for ICD implantation to Class IIa. At 9-month follow-up, no arrhythmic events occurred. This case exemplifies how CMR uniquely captures myocardial fibrosis burden, now recognized as an essential substrate for malignant Vas, and demonstrates its growing role in reshaping clinical decisions in patients who would otherwise remain unprotected by conventional assessment.

The eight contributions assembled in this Special Issue collectively delineate the evolving landscape of VA management across heterogeneous clinical settings, from channelopathies and cardiomyopathies to post-surgical congenital heart disease and ischemic heart disease. Taken together, they reinforce a central clinical imperative: careful, individualized management of VAs is fundamental to improving patient outcomes and reducing the burden of SCD.

A recurring theme across contributions is the need for refined risk stratification beyond conventional tools. Left ventricular ejection fraction, QTc interval, and symptom burden, while clinically relevant, are insufficient to identify all patients at meaningful risk of life-threatening arrhythmias. The contributions presented here highlight three complementary pillars of enhanced risk stratification. First, novel biomarkers such as apelin may capture neurohormonal perturbations preceding clinically manifest arrhythmia, offering a window for preventive intervention in HFrEF (Contribution 1). Second, advanced echocardiographic techniques, specifically speckle-tracking strain imaging, provide more sensitive and specific myocardial deformation assessment than conventional functional parameters, as demonstrated by the superiority of RVFWLS in ARVC (Contribution 3). Third, multiparametric CMR has emerged as an indispensable tool for SCD risk stratification in structural heart disease (Contribution 8). Its ability to quantify fibrosis burden through LGE and detect diffuse interstitial remodeling through T1 mapping and ECV fundamentally transforms clinical decision-making, enabling ICD implantation in patients who would otherwise remain unprotected.

The importance of disease-specific management protocols is also underlined by this collection. TTS has historically been viewed as a transient and largely benign cardiomyopathy [8,9]; however, Contribution 2 demonstrates that early VAs in TTS confer a nearly fourfold increase in long-term adverse cardiac events, independent of hemodynamic recovery. This mandates systematic arrhythmia evaluation before discharge and individualized follow-up in affected patients. In rTOF, the progressive evolution of arrhythmogenic substrate across decades demands lifelong surveillance and timely electrophysiological intervention (Contribution 4). The emerging proactive ablation paradigm represents a fundamental reconceptualization of the therapeutic objective: from treating arrhythmia after it occurs to eliminating substrate before clinical manifestation, particularly in the pre-pulmonary valve replacement setting where subsequent transcatheter intervention may close the therapeutic window.

The contributions also highlight significant advances in therapeutic strategies. Catheter ablation remains the cornerstone of invasive VA management; however, a meaningful subset of patients present with refractory VT for whom no further options previously existed. For this population, STAR has emerged as a transformative though still investigational approach, overcoming the anatomical and hemodynamic limitations of conventional ablation by achieving myocardial fibrosis non-invasively [10] (Contributions 5 and 7). The integration of noninvasive electrocardiographic imaging further enables a fully non-invasive workflow, as illustrated in Contribution 7, where LV apical thrombosis precluded any invasive approach. Nevertheless, STAR remains a palliative treatment reserved for patients without viable alternatives, and rigorous patient selection within specialized multidisciplinary teams is essential. Device therapy also continues to evolve: the EV-ICD presented in Contribution 6 addresses longstanding limitations of transvenous devices in pediatric patients with inherited arrhythmia syndromes, where somatic growth and venous access preservation are critical long-term considerations.

The management of VAs demands precision in diagnosis, risk stratification, and therapy selection. The contributions of this Special Issue exemplify the multidisciplinary convergence of imaging, electrophysiology, genetic medicine, radiation oncology, and device engineering now required to optimize outcomes in this complex patient population. As the field continues to advance, clinicians must integrate these evolving tools into patient-centered decision frameworks, recognizing that the greatest gains in survival and quality of life will emerge from their thoughtful and individualized combination.
